# Minor allele of the factor V K858R variant protects from venous thrombosis only in non-carriers of factor V Leiden mutation

**DOI:** 10.1038/s41598-019-40172-x

**Published:** 2019-03-06

**Authors:** M. Ibrahim-Kosta, P. Suchon, F. Couturaud, D. Smadja, R. Olaso, M. Germain, N. Saut, L. Goumidi, C. Derbois, F. Thibord, S. Debette, P. Amouyel, J. F. Deleuze, P. van Doorn, E. Castoldi, E. Patin, M. C. Alessi, D. A. Trégouët, P. E. Morange

**Affiliations:** 1 Laboratory of Haematology, La Timone Hospital, Marseille, France; 2 C2VN, Aix Marseille Univ, INSERM, INRA, C2VN Marseille, France; 3 University Brest, France, Department of Chest Diseases and Internal Medicine, Hôpital de la Cavale Blanche, Brest, France; 4 Service d’hématologie biologique, AP-HP, Hôpital Européen Georges Pompidou, Paris, France; 5 Université Paris Descartes, Sorbonne Paris Cité, France, Inserm, UMR-S1140 Paris, France; 6 Centre National de Recherche en Génomique Humaine, Direction de la Recherche Fondamentale, CEA, Evry, France; 7 INSERM UMR_S 1219, Bordeaux Population Health Research Center, University of Bordeaux, Bordeaux, France; 8 CRB Assistance Publique - Hôpitaux de Marseille, HemoVasc (CRB AP-HM HemoVasc), Marseille, France; 9 Sorbonne-Université, Pierre Louis Doctoral School of Public Health, Paris, France; 10 Department of Neurology, Bordeaux University Hospital, Bordeaux, France; 11 Univ. Lille, INSERM, Centre Hosp. Univ Lille, Institut Pasteur de Lille, LabEx DISTALZ-UMR1167 - RID-AGE - Risk factors and molecular determinants of aging-related diseases, Epidemiology and Public Health Department, F-Lille, France; 12 CEPH, Fondation Jean Dausset, Paris, France; 13 Department of Biochemistry, Cardiovascular Research Institute Maastricht, Maastricht University, Maastricht, The Netherlands; 14 Human Evolutionary Genetics Unit, Department of Genomes & Genetics, Institut Pasteur, Paris, France; 15 CNRS, UMR2000 Paris, France; 16 Center of Bioinformatics, Biostatistics and Integrative Biology, Institut Pasteur, Paris, France

## Abstract

Factor V serves an important role in the regulation of blood coagulation. The rs6025 (R534Q) and rs4524 (K858R) polymorphisms in the *F5* gene, are known to influence the risk of venous thrombosis. While the rare Q534 (factor V Leiden) allele is associated with an increased risk of venous thrombosis, the minor R858 allele is associated with a lower risk of disease. However, no study has deeply examined the cumulative impact of these two variations on venous thrombosis risk. We study the association of these polymorphisms with the risk of venous thrombosis in 4 French case-control populations comprising 3719 patients and 4086 controls. We demonstrate that the Q534 allele has a dominant effect over R858. Besides, we show that in individuals not carrying the Q534 allele, the protective effect of the R858 allele acts in a dominant mode. Thrombin generation-based normalized activated protein C sensitivity ratio was lower in the 858R/R homozygotes than in the 858K/K homozygotes (1.92 ± 1.61 vs 2.81 ± 1.57, *p* = 0.025). We demonstrate that the R858 allele of the *F5* rs4524 variant protects from venous thrombosis only in non-carriers of the Q534 allele of the *F5* rs6025. Its protective effect is mediated by reduced factor VIII levels and reduced activated protein C resistance.

## Introduction

Factor V (FV) serves an important role in the regulation of blood coagulation, having both pro- and anticoagulant properties^1^. FV circulates in blood as a precursor of activated FV (FVa), which serves as a cofactor to FXa in the activation of prothrombin to thrombin. The procoagulant activity of FVa is under strict control by activated protein C (APC), which cleaves multiple peptide bonds in FVa^2^. FV also has two identified anticoagulant activities, as a cofactor to APC in the inactivation of factor VIIIa^3^ (FVIIIa) and as a cofactor to the coagulation inhibitor tissue factor pathway inhibitor (TFPIα) in the inhibition of FXa^4,5^.

A large number of missense polymorphisms in the *F5* gene coding for FV has been reported^6^. Among these, two genetic variations are now well established to affect the risk of venous thrombosis (VT): FV Leiden (FVL, rs6025, R534Q) identified by Bertina *et al*.^7^ and the Lysine to Arginine substitution at amino acid 858 (rs4524, K858R) identified by Smith *et al*.^8^. The Q534 allele is the major genetic risk factor of VT and has a frequency of ∼5% in the general population of European descent. The Q534 allele has been associated with a ∼3 fold increased risk of VT through resistance to APC^9^. Conversely, the minor R858 allele of rs4524 has been associated with a protective OR (∼0.8) for VT^8^. This association has been replicated in other studies and meta-analyses^10–14^. There is some evidence that the protective effect of the R858 allele is mediated through its influence on plasma APC resistance^15,16^.

Although several studies have previously attempted to address the joint influence of the rs6025 and rs4524 polymorphisms on VT risk^10,12,14,15^, none of them have properly taken into account the linkage disequilibrium (LD) between the two polymorphisms to accurately estimate their respective influence on disease risk. Indeed, haplotype analysis is not only adapted to detect interactive effects between polymorphisms^17,18^ but is also particularly well suited to identify the true contribution on a trait of a polymorphisms from what is due to its LD with other variant(s)^19,20^.

In this work, we performed a comprehensive haplotype analysis of the rs6025 and rs4524 in a case-control setting totaling 3716 VT patients and 4086 controls in order to better estimate their true impact on VT risk. In addition, we supplemented our epidemiological observations with experimental data on the functional impact of the rs4524 on the PC anticoagulant pathway.

## Results

### Association of rs6025 (R534Q) and rs4524 (K858R) variants with VT

Genotype distributions of the two studied *F5* variants in cases and controls are provided in Table 1. As already well documented, the presence of the Q534 allele was about 3-fold more frequent in cases than in controls (0.083 vs 0.025, *p* = 4.37 10^−63^). Conversely, the minor allele (R858) of the rs4524 variant was less frequent in cases than in controls (0.213 vs 0.259, *p* = 2.14 10^−11^). The Q534 allele was associated with an increased risk of VT (OR = 3.61 [3.06–4.24]), while the R858 was associated with a decreased risk of VT (OR = 0.77 [0.72–0.84]) in the combined study samples with no evidence for heterogeneity across studies (*p* = 0.778).

**Table 1:** Association of *F5* rs6025 and rs4524 with VT risk in four French case-control studies.

	rs6025 (R534Q)				rs4524 (K858R)			
	R/R	R/Q	Q/Q	*P* value^a^	K/K	K/R	R/R	*P* value^a^
	FVL^−^/FVL^−^	FVL^+^/ FVL^−^	FVL^+^/ FVL^+^					
EDITH								
Controls	1103 (95%)	56 (5%)	1	8.79 10^−10^	656 (56%)	445 (38%)	69 (6%)	0.0077
Cases	1030 (88%)	138 (12%)	3		704 (61%)	396 (34%)	51 (5%)	
EOVT								
Controls	1170 (95%)	58 (5%)	0	1.72 10^−16^	672 (55%)	477 (39%)	79 (6%)	0.010
Cases	340 (83%)	70 (17%)	1		255 (62%)	136 (33%)	20 (5%)	
FARIVE								
Controls	561 (95%)	27 (5%)	0	8.43 10^−5^	314 (54%)	220 (38%)	44 (8%)	0.0031
Cases	532 (89%)	62 (11%)	1		363 (63%)	184 (32%)	31 (5%)	
MARTHA								
Controls	1052 (95%)	58 (5%)	0	5.26 10^−23^	586 (53%)	460 (41%)	64 (6%)	3.19 10^−6^
Cases	1202 (68%)	340 (22%)	0		973 (63%)	490 (32%)	79 (5%)	
COMBINED								
Controls	3886 (95%)	199 (~5%)	1	4.37 10^−63^	2228 (55%)	1602 (39%)	256 (6%)	2.14 10^−11^
Cases	3104 (83%)	610 (16%)	5 (1‰)		2295 (62%)	1206 (33%)	181 (5%)	

^a^Cochran Armitage trend test’s *p*-value.

FVL^−^: absence of Factor V Leiden mutation; FVL^+^: presence of Factor V Leiden mutation.

### Linkage disequilibrium and haplotype analyses of the rs6025 (R534Q) and rs4524 (K858R) variants

The two variants were in complete negative (*D’* = −1) LD, generating 3 haplotypes: R534/K858 (H1), Q534/K858 (H2) and R534/R858 (H3). Haplotype distributions in cases and controls are shown in Table 2. These distributions were very consistent across the four studies. In agreement with the results of the single variant analyses, the unique haplotype (H2) carrying the Q534 allele was more frequent in cases than in controls, whereas the unique haplotype (H3) carrying the R858 form was less frequent in cases, homogeneously across the four studies.

**Table 2:** Association of haplotypes derived from *F5* rs6025 (R534Q) and rs4524 (K858R) with VT risk.

		Controls	Cases	OR (95%CI)
**EDITH**		**(N** = **1150)**	**(N** = **1140)**	
H1	R534/K858	0.73	0.73	—
H2	Q534/K858	0.02	0.06	2.58 [1.85–3.61]
H3	R534/R858	0.25	0.21	0.85 [0.74–0.98]
**EOVT**		**(N** = **1228)**	**(N** = **411)**	
H1	R534/K858	0.72	0.70	—
H2	Q534/K858	0.02	0.09	3.51 [2.38–5.17]
H3	R534/R858	0.26	0.21	0.86 [0.70–1.04]
**FARIVE**		**(N** = **575)**	**(N** = **577)**	
H1	R534/K858	0.71	0.73	—
H2	Q534/K858	0.02	0.06	2.26 [1.41–3.61]
H3	R534/R858	0.27	0.21	0.78 [0.65–0.95]
**MARTHA**		**(N** = **1110)**	**(N** = **1542)**	
H1	R534/K858	0.71	0.68	—
H2	Q534/K858	0.03	0.11	4.96 [3.67–6.71]
H3	R534/R858	0.26	0.21	0.83 [0.73–0.95]

### Association of F5 diplotypes with VT

Because of the strong LD between the two *F5* variants, the three observed haplotypes generated 5 diplotypes, i.e pairs of haplotypes carried by a given individual. Association of these diplotypes with VT risk in the combined studies are shown in Table 3. In the absence of the Q534 allele, carrying one or two copies of the H3 haplotype was homogeneously associated with a protective OR for VT, OR = 0.78 [0.70–0.88] and OR = 0.74 [0.58–0.94], respectively. The test for heterogeneity between these two ORs was not significant (*p* = 0.68) indicating a dominant effect of the H3 haplotype in non carriers of the Q534 allele

**Table 3:** Distribution of diplotypes derived from *F5* rs6025 (R534Q) rs4524 (K858R) variants in the combined cases and control population.

Diplotype		Controls	Cases	
H1	H1	2064 (51%)	1812 (49%)	Reference
H1	H2	146 (4%)	474 (13%)	OR = 2.99 [2.36–3.79] *p* = 1.58 10^–19^
H2	H3	53 (1%)	131 (4%)	OR = 2.36 [1.61–3.45] *p* = 1.06 10^–5^
H2	H2	0	5 (0, 1%)	NA
H1	H3	1545 (38%)	1071 (29%)	OR = 0.78 [0.70–0.88] *p* = 5.97 10^−5^
H3	H3	255 (6%)	178 (5%)	OR = 0.74 [0.58–0.94] *p* = 0.013

H1 haplotype refers to the R534/K858 haplotype. H2 haplotype represents the unique haplotype carrying the FVL mutation (Q534/K858). H3 haplotype represents the unique haplotype carrying the rare R858 allele (R534/R858).

Odds Ratios (OR) were adjusted for age, sex and study population.

By contrast, we observed a dominant effect of the H2 haplotype (tagging for the Q534 allele), as H2 carriers were exposed to the same VT risk whether they were carrying the H1H2 or the H2H3 diplotype, OR = 2.99 [2.36–3.79] and OR = 2.36 [1.61–3.45], respectively (*p* for heterogeneity = 0.30).

### Association of F5 diplotypes with quantitative biological phenotypes

Association of *F5* diplotypes with plasma levels of FV and normalized Agkistrodon Contortrix Venom ratio (ACVn) from MARTHA GWAS and MARTHA12 cases are shown in Table 4. Association analyses for ACVn were conducted after excluding individuals on anticoagulant therapy. We observed a significant association between ACVn and the H2 haplotype (tagging for the Q534 allele: *p* = 1.79 10^−169^ for H1H2 and *p* = 1.00 10^−59^ for H2H3). Carrying a diplotype including the H3 haplotype (tagging for R858) in the absence of the Q534 allele had no effect on the quantitative biological phenotypes measured (ACVn and FV plasma levels). Only a trend toward an association between H3 haplotype and lower APC resistance in the ACVn test was observed under a dominant model (*p* = 0.06).

**Table 4:** Association of *F5* diplotypes with quantitative biological phenotypes in MARTHA GWAS and MARTHA 12.

Diplotype	Log ACVn*			FV plasma levels (IU/mL)		
	n		*p*	n		*p*
H1H1	450	0.051 (0.235)	Ref	480	1.09 (0.236)	Ref
H1H2	87	−0.723 (0.210)	1.79 10^−169^	98	1.09 (0.261)	0.42
H2H3	29	−0.700 (0.193)	1.00 10^−59^	31	1.13 (0.268)	0.20
H1H3	281	0.072 (0.261)	0.14	293	1.07 (0.210)	0.18
H3H3	39	0.096 (0.244)	0.20	42	1.05 (0.149)	0.53

H1 haplotype refers to the R534/K858 haplotype. H2 haplotype represents the unique haplotype carrying the FV Leiden mutation (Q534/K858). H3 haplotype represents the unique haplotype carrying the rare R858 allele (R534/R858).

ACVn: normalized Agkistrodon Contortrix Venom ratio; FV: Factor V.

Association analyses were adjusted for age, sex and MARTHA substudy group. *Analyses were conducted after excluding individuals under anticoagulant therapy.

**Figure 1:**
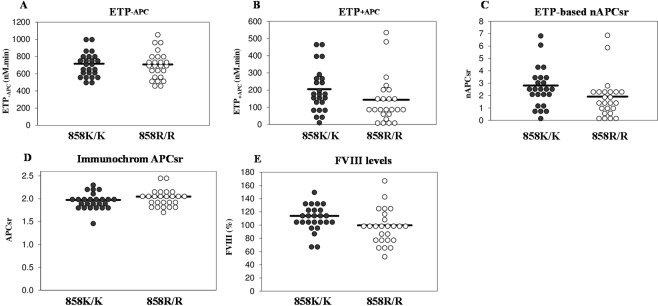
Coagulation parameters in 858K/K and 858R/R homozygotes. Distributions of the ETP^−APC^ (**A**), ETP^+APC^ (**B**) and nAPCsr (**C**) measured with thrombin generation-based assay, the APCsr measured with the Immonochrom assay (**D**), and FVIII levels (**E**) in 25 homozygous carriers of rs4524 (858R/R) and in 25 non-carriers (858K/K). The horizontal lines represent the means of the distributions. Please note that APC resistance increases with increasing ETP-based nAPCsr and with decreasing Immunochrom APCsr.

To get more insight into the protective effect of the rs4524 R858 allele, we measured thrombin generation at 10 pM tissue factor (TF) in the absence and presence of APC in 25 homozygous carriers of this allele (858R/R) and in 25 non-carriers (858K/K), all without Q534 allele (Fig. 1). While the area under the thrombin generation curve without APC (endogenous thrombin potential (ETP)^−APC^, Fig. 1A) was similar in the two genotype groups (707.8 ± 158.3 nM.min vs 716.0 ± 139.1 nM.min, *p* = 0.876 after adjustment for age and sex), the ETP plus APC (ETP^+APC^, Fig. 1B) was lower in the 858R/R homozygotes than in the 858K/K homozygotes (143.4 ± 133.3 nM.min vs 205.2 ± 127.7 nM.min) and the difference was close to significance after correction for age and sex (*p* = 0.067). Accordingly, the normalised APC sensitivity ratio (nAPCsr, Fig. 1C) was also lower in the 858R/R homozygotes than in the 858K/K homozygotes (1.92 ± 1.61 vs 2.81 ± 1.57), with *p* = 0.025. This indicates that the minor allele of the rs4524 polymorphism is associated with reduced plasma APC resistance in the ETP-based assay, in line with its protective effect against VT.

In contrast, no significant difference in APC sensitivity ratio (APCsr) between 858R/R and 858K/K homozygotes was observed in the Immunochrom assay (2.05 ± 0.18 vs 1.97 ± 018, *p* = 0.169 after correction for age and sex) (Fig. 1D), which specifically measures APC resistance arising from poor FVIIIa inactivation. However, FVIII levels (Fig. 1E) were somewhat lower in 858R/R homozygotes than in 858K/K homozygotes (99.7 ± 26.4 IU/dL vs 113.9 ± 19.4 IU/dL, *p* = 0.039).

## Discussion

In the present study, we performed the first haplotype/diplotype association analysis of the *F5* rs6025 and rs4524 polymorphisms with respect to VT risk. This analysis that efficiency takes into account the LD between the two polymorphisms for accurately estimating their true impact on VT risk, demonstrated that the R858 allele of the rs4524 is protective against VT but only in individuals not carrying the Q534 allele of the rs6025. Conversely, the Q534 allele is a risk factor for VT whatever the allele present at the rs4524 locus.

Due to a complete negative LD between the 2 variants, the R858 and the Q534 alleles are never on the same haplotype in the French studied populations.

Whereas the mechanism by which the Q534 allele induces a hypercoagulable state is well documented^1^, the mechanisms underlying the protection from VT provided by the R858 allele deserve to be clarified. The K858R variant is located in the N-terminal part of the B-domain and is part of the so-called G-allele, a particular *F5* allele which is characterized by having guanines (G) instead of adenines (A) at nucleotide positions 2391, 2663 (rs4524), 2684 and 2863^6^. These variants are always co-inherited in Caucasians and the last three lead to amino acid substitutions. However, not much is known about the functional effects of most of the B-domain variants. Since the B-domain is removed upon activation of FV, an effect on the activity of FVa seems unlikely, but an effect on the anticoagulant functions of FV, when the B-domain is retained, cannot be excluded. In fact, the FV B-domain is known to be important for the APC-cofactor function of FV in the inactivation of FVIIIa^21^. In this respect, Kostka *et al*.^15^ have shown that the R858 allele is less frequent than expected in patients with APC resistance in the absence of the Q534 allele. In this study, APC resistance was measured with the Immunochrom® APC response Test Kit, which specifically measures the APC-cofactor activity of FV in FVIIIa inactivation. In addition, Mingozzi *et al*.^16^ have observed that the R858 allele is associated with lower APC resistance (measured with the activated partial thromboplastin time aPTT-based assay) in asymptomatic Q534 heterozygotes. In the present study we did not observe any association between the H3 haplotype (tagging the R858 allele) and the ACVn, an aPTT-based APC resistance assay, nor with FV plasma levels. Moreover, the effect of the H2 haplotype (tagging for the Q534 allele) on APC resistance was similar irrespective of the haplotype (H1 or H3) on the counterpart allele.

To get more insight into the biological mechanisms responsible for the R858 allele, additional functional assays exploring the PC anticoagulant pathway were performed in 25 homozygous carriers of R858 and in 25 non-carriers. While we could not prove that R858/R858 homozygotes are less APC-resistant than K858/K858 homozygotes in the Immunochrom test (probably due to the insufficient number of patients tested in relation to the ‘assay window’, which is very narrow), we did observe reduced APC resistance in R858/R858 homozygotes using the ETP-based assay, essentially confirming that the R858 variant attenuates APC resistance. In addition, we found that FVIII levels are ~13% lower in R858/R858 homozygotes than in K858/K858 homozygotes, which could contribute to the protective effect of the R858 allele.

Apart from APC resistance, it has been recently reported that the B-domain of FV is also important for the interaction with TFPIα^22^ and that it can be alternatively spliced to yield a form of FV (FV-short) with considerably increased affinity for TFPIα^23^. Therefore, it is tempting to speculate that the rs4524 polymorphism might also affect the FV-TFPIα interaction, thereby influencing the TFPIα-cofactor activity of FV in the inhibition of FXa^4,5^ and/or the inhibition of FV activation and prothrombinase by TFPIα^22–24^. This could also explain why the effect of K858R on APC resistance is more easily detected with the ETP-based assay (which is triggered with tissue factor and is very sensitive to TFPIα^25^ than with aPTT-based assays, which rely on the intrinsic coagulation pathway.

In conclusion, we have provided additional evidence that the common R858 variant protects against VT and that its protective effect is mediated by reduced FVIII levels and reduced APC resistance. Additional functional studies are needed to clarify whether this polymorphism also affects the interaction of FV with TFPIα and whether this interaction contributes to the protective effect of R858 on VT.

## Materials and Methods

The present work was based on 4 case-control studies for VT, namely EDITH, EOVT, FARIVE, and the MARTHA Genome-Wide Association Study (GWAS), where VT events (pulmonary embolism and/or deep vein thrombosis) were objectively diagnosed. Detailed descriptions have already been published^13,26^ and are summarized in the supplementary text. The association of FV diplotypes with quantitative phenotypes was assessed in two independent collections of VT cases, the MARTHA GWAS and the MARTHA12^13,27^ study (described in the supplementary text).

Participants of the case-control EOVT and MARTHA GWAS studies have been typed with high density Illumina DNA arrays and imputed for 1000G reference database as part of previous Genome-Wide Association Studies^13,27^. In both studies, the rs6025 and rs4524 were well imputed (imputation criterion *r*^2^ > 0.80). In the FARIVE and EDITH studies, the rs6025 and rs4524 polymorphisms were genotyped by Taqman Technology (Applied Biosystems C___ 11975250_10 and C___8919444_1 respectively). MARTHA12 participants were typed with the Illumina HumanExome BeadChip v1.0 that includes both the rs6025 and rs4524.

All patients have been informed and their consents have been obtained.

The procedures employed were reviewed and approved by the *Assistance Publique des Hopitaux de Marseille* institutional review committee. All methods were performed in accordance with the relevant guidelines and regulations.

### Functional assays

In MARTHA GWAS and MARTHA12, fasting blood was drawn and biological parameters measured in platelet-poor plasma. Using those plasma, we performed several experiments in order to assess the functional impact of the rs4524 on the PC anticoagulant pathway by different assays. The ACV test is a global aPTT-based test exploring functional defects in the PC anticoagulant pathway. In MARTHA patients free from any anticoagulant treatment, the ACV test was performed as described by Robert *et al*.^28,29^. Briefly, the results were expressed as ACV ratio (ACVr) which was obtained by dividing the aPTT plus ACV (Protac^®^) by the aPTT. ACVn was then calculated by dividing the ACVr of the patient by the ACVr of a normal plasma control. FV plasma levels were measured by a clotting-based assay using an automated coagulometer (STA-R, Diagnostica Stago).

Among the cases of MARTHA12 that were not on anticoagulant treatment at the time of blood collection, 25 homozygous carriers of the minor allele of the rs4524 polymorphism (858R/R) and 25 sex-matched non-carriers (858K/K) were selected for functional assays. None of the selected individuals carried FVL (i.e. all were R534/R534).

For these selected patients, thrombin generation at 10 pM TF in the absence and presence of APC was measured using the Calibrated Automated Thrombography (CAT) method^30^. The area under the thrombin generation curve (in nM.min), calculated by the Thrombinoscope software, was used as the main output parameter. The APC concentration (7 nM) was chosen such as to reduce the ETP of normal pooled plasma to ~10% of its value in the absence of APC (%rest = ETP^+APC^/ETP^−APC^ = 10%). The nAPCsr was calculated by dividing the %rest of each sample plasma by the %rest of normal pooled plasma measured on the same plate. The nAPCsr varies between 0 and 10 and is directly correlated with APC resistance.

The Immunochrom APC Resistance assay, which specifically detects APC resistance arising from poor FVIIIa inactivation, was carried out as described in Brugge *et al*.^31^. This assay is based on the chromogenic measurement of FVIIIa activity before and after a standardised treatment of the (diluted) sample plasma with APC. The assay outcome is expressed as APCsr, which is defined as the ratio between the FVIIIa activity determined in the absence and presence of APC. The Immunochrom APCsr is inversely correlated with APC resistance. All samples were measured in duplicate.

### Statistical analysis

The association of *F5* genotypes with VT risk was tested using the Cochran-Armitage trend test after having checked for the genotype distributions consistency with Hardy-Weinberg equilibrium. Analyses were performed separately in each study and results were then meta-analyzed using the Mantel-Haensel methodology implementing a fixed effect model. Due to the complete negative (*D’* = −1) linkage disequilibrium between the *F5* variants, haplotypes and diplotypes could be manually reconstructed from genotypes. The statistical significance of the linkage disequilibrium D’ coefficient was assessed by the maximum likelihood approach implemented in the THESIAS software^32^ that relies on the methodology proposed by Thompson *et al*. 1988^33^. Associations of haplotypes/diplotypes with VT risk and quantitative phenotypes were tested using a generalized linear model and were adjusted for age, sex and study group. Heterogeneity across populations was tested using the Cochran’s Q statistic.

Coagulation parameters measured in plasma were compared between carriers and non-carriers of the rs4524 polymorphism using linear regression analysis adjusted for age and sex.

## Supplementary information

supplementary data
