# Supplementary material for: Minor allele of the factor V K858R variant protects from venous thrombosis only in non-carriers of factor V Leiden mutation
Source: Sci Rep. 2019 Mar 6;9:3750. doi: 10.1038/s41598-019-40172-x (PMC6403374; doi:10.1038/s41598-019-40172-x)
Supplement: Supplementary file 1 — supplementary data [file 41598_2019_40172_MOESM1_ESM.pdf]

Supplementary data

Minor allele of the factor V K858R variant protects from venous thrombosis only in non-carriers of factor V Leiden mutation

Ibrahim-Kosta M[1,2], Suchon P[1,2], Couturaud F[3], Smadja D [4], Olaso R[5], Germain M[6], Saut N[1,2,7], Goumidi L[2], Derbois C[5], Thibord F[6], Debette S[8], Amouyel P[9], Deleuze JF[5,10], van Doorn P[11], Castoldi E[11], Patin E[12,13,14], Alessi MC[1,2], Trégouët DA[6], Morange PE[1,2,7].

1 Laboratory of Haematology, La Timone Hospital, Marseille, France.

2 C2VN, Aix Marseille Univ, INSERM, INRA, C2VN, Marseille, France.

3 University, 29200 Brest, France; Department of Chest Diseases and Internal Medicine, Hôpital de la Cavale Blanche, 29609 Brest Cedex, France

4 Service d'hématologie biologique, AP-HP, Hôpital Européen Georges Pompidou, Paris, France. Université Paris Descartes, Sorbonne Paris Cité, France, Inserm UMR-S1140, Paris, France

5 Centre National de Recherche en Génomique Humaine, Direction de la Recherche Fondamentale, CEA, 91057 Evry, France

6 Institut National pour la Santé et la Recherche Médicale (INSERM), Unité Mixte de Recherche en Santé (UMR_S) 1166, 75013 Paris, France ; Sorbonne Universités, Université Pierre et Marie Curie (UPMC Univ Paris 06), UMR_S 1166, Team Genomics & Pathophysiology of Cardiovascular Diseases, 75013 Paris, France; Institute for Cardiometabolism and Nutrition (ICAN), 75013 Paris, France.

7 CRB Assistance Publique - Hôpitaux de Marseille, HemoVasc (CRB AP-HM HemoVasc), Marseille, France.

8 INSERM UMR_S 1219, Bordeaux Population Health Research Center, University of Bordeaux, France; Department of Neurology, Bordeaux University Hospital, Bordeaux, France.

9 Univ. Lille, INSERM, Centre Hosp. Univ Lille, Institut Pasteur de Lille, LabEx DISTALZ-UMR1167 - RID-AGE - Risk factors and molecular determinants of aging-related diseases, Epidemiology and Public Health Department, F-59000 Lille, France.

10 CEPH, Fondation Jean Dausset, Paris, France.

11 Department of Biochemistry, Cardiovascular Research Institute Maastricht, Maastricht University, Maastricht, The Netherlands.

12 Human Evolutionary Genetics Unit, Department of Genomes & Genetics, Institut Pasteur, Paris 75015, France.

13 CNRS UMR2000, Paris 75015, France.

14 Center of Bioinformatics, Biostatistics and Integrative Biology, Institut Pasteur, Paris 75015, France.

- Trégouët DA and Morange PE contributed equally to this work.

- EDITH [Oger E *et al* 2006]: The EDITH study is a case-control study that was designed to test interactions between genetic and environmental risk factors of VT. Between May 2000 and December 2009, all consecutive unselected in- and outpatients seen at Brest (West of France) University Hospital for symptomatic VT were asked to participate in the study. Controls were selected from the roster of patients hospitalized in the same ward in the following 12 months after the case's event date. Controls were not included if they had a past history of VT or if they were receiving long-term anticoagulant therapy.

- EOVT [Tregouet DA et al 2009]: The EOVT study is composed of 453 patients with early age of onset of VT (<50 years) recruited in 4 different French centers (Grenoble, Marseille, Montpellier, and Paris) between 1999 and 2006, and 1327 healthy French persons from the Suvimax study. Criteria for patient inclusion in the study were (1) European origin; (2) early age of onset of first VTE (<50 years); (3) Deep VT and/or pulmonary embolism as first event (documented by venography, Doppler ultrasound, angiography, and/or ventilation/perfusion lung scan); (4) lack of acquired risk factors at the time of VT (including surgery, hospitalization, pregnancy, puerperium, oral contraception, cancer, and autoimmune disease); and (5) lack of strong known genetic risk factors, including antithrombin, protein C or protein S deficiencies, and homozygosity for FV Leiden or Factor II 20210A. Criteria for inclusion of the 1327 studied controls were European origin, no chronic conditions, and no regular medicines.

- FARIVE [Tregouet DA *et al* 2009]: The FARIVE study is a multicenter case-control study of 607 patients with a first episode of proximal deep VT and/or pulmonary embolism. Patients younger than 18 years, with previous VT event, that had a diagnosis of active cancer or a history of malignancy less than 5 years previously, or have a short life expectancy because of other causes, were excluded. The control group consists of age- and sex-matched individuals free of venous and arterial thrombotic disease. Potential control subjects with cancer, liver or kidney failure, or a history of venous and/or arterial thrombotic disease are ineligible.

- MARTHA: MARTHA Genome Wide Association Studies (GWAS) study has been extensively described in Oudot-Mellakh T *et al* 2012, Antoni G *et al* 2010 and Germain M *et al* 2012. All patients were free of any chronic conditions and free of any well characterized genetic risk factors including antithrombin, protein C or protein S deficiency, homozygosity for FV Leiden or Factor II 20210A, and lupus anticoagulant. The control group was composed of 3,690 healthy subjects selected from the Paris Prospective Study 3 (PPS3) [Empana *et al* 2015].

- MARTHA12 [Germain *et al* 2015]: The MARTHA12 study is composed of an independent sample of 1,245 VT patients. Patients have been recruited between 2010 and 2012 according to the same criteria as the MARTHA patients.
